# The perception of e-learning during the SARS-CoV-2 pandemic by students of medical universities in Poland – a survey-based study

**DOI:** 10.1186/s12909-022-03600-7

**Published:** 2022-07-08

**Authors:** Nicola Dyrek, Agnieszka Wikarek, Małgorzata Niemiec, Aleksander J. Owczarek, Magdalena Olszanecka-Glinianowicz, Piotr Kocełak

**Affiliations:** 1 Students’ Scientific Society at the Pathophysiology Unit, Department of Pathophysiology, Faculty of Medical Sciences in Katowice, the Medical University of Silesia, Katowice, Poland; 2 Health Promotion and Obesity Management Unit, Department of Pathophysiology, Faculty of Medical Sciences in Katowice, the Medical University of Silesia, Katowice, Poland; 3 Pathophysiology Unit, Department of Pathophysiology, Faculty of Medical Sciences in Katowice, the Medical University of Silesia, Medyków Street 18, 40-752 Katowice, Poland

**Keywords:** E-learning, SARS-CoV-2, The pandemic

## Abstract

**Background:**

In March 2020 lockdown due to the COVID-19 pandemic forced Polish Medical Universities to implement e-learning. The aim of the study was to evaluate the perception of e-learning by students of Medical Universities in Poland.

**Material and methods:**

Survey was performed nationwide via the Internet from 30th November 2020 to 10th February 2021. Six hundred fifteen (615) medical students completed the survey. The study questionnaire included questions concerning sociodemographic data, perception of lecturers’ effectiveness, assessment of stationary and online classes, changes in learning habits and restrictions on education, and advantages and disadvantages of e-learning.

**Results:**

The respondents reported that 96.1% of lectures, 85.5% of seminars, and 40.0% of clinical classes were implemented by e-learning. The lectures conducted by e-learning were assessed as good and very good by 78.4% and seminars by 51.2% of respondents. While the clinical classes conducted by e-learning were assessed as bad and very bad by 62.9% of respondents.

The most frequently indicated limitations of e-learning were the quality of the content and available materials (26.9%), restrictions in direct contact with the lecturer (19.6%), Internet connection (16.8%), and home conditions (13.8%). Only 4% of the students had to buy or retrofit computer equipment. Any other limitations were indicated by 9.7% of the respondents.

**Conclusions:**

Students were highly accepting of lectures and seminars conducted in the form of e-learning, but not laboratory and clinical classes. The main problems in e-learning are the quality of the classes conducted and the Internet connection. The students expect e-learning classes to be conducted in real-time, with direct, face-to-face contact with the lecturer.

## Introduction

The COVID-19 pandemic is a surprising phenomenon in modern history. For 100 years there has not been a similar epidemiological event until today [1]. To reduce the number of infections, many countries decided to introduce a lockdown, including all levels of the education system. By and large, traditional education has been replaced by various forms of e-learning. It should be noted that the trends of partial replacement of traditional education by e-learning have been performed before [2, 3] and the pandemic accelerated that process. However, during the introduction of lockdown, the experiences of Medical Universities with e-learning were limited [4]. Before the pandemic, Polish Universities used e-learning for carrying out single lectures and seminars. After the introduction of lockdown, the University authorities and lecturers had to make a great effort to meet the changing education system. It was especially difficult at the Medical Universities due to their specific need for patient contact during practical instruction. Clinical hospitals were overcrowded and there was no space, time, or protection equipment to ensure the safety of patients and students. Both lecturers and students stood in front of an arduous task. Students had to motivate themselves to work more independently and get used to the lack of contact with lecturers and patients. Whereas many lecturers were not prepared to be used in the teaching process of e-learning platforms, or applications such as MS TEAMS, ZOOM, and others. Thus, lecturers have been spending a lot of valuable time becoming familiar with e-learning methods, which was especially difficult for clinicians burdened with patient care. Support from information technology (IT) staff was very important but it has also not ensured avoiding problems associated with the Internet connection [5]. Consequently, classes were prolonged, or not all material was realized. Furthermore, some students could not attend the classes due to technical problems. Moreover, most of the students returned to their own homes, which resulted in disruption of learning by household members or the lack of a place to study [4].

The most challenging and concerning issue in e-learning and hybrid learning is to provide a good standard of practical experiences for medical students. It should be noted that the core of medical education constitutes a model of gaining practical experiences [6]. Some of the students volunteered to help health care staff with basic hospital tasks. Although they have acquired a lot of experience, they have not completed appropriate practical classes that cannot be made up at this time due to the limitations of precepting students and staff shortages. Many concerns have been raised that insufficient theoretical background and psychological stress of medical students can negatively affect their education. Indeed, moderate, or extreme levels of stress during the pandemic were observed in 82% of Chinese medical students [7] and 54.5% of Irish medical students [8]. It has been found that the main sources of stress are the risk of infection, the possibility of transmission of the disease to relatives, and contact with other students or patients in hospitals [8, 9], as well as social isolation and loneliness [10]. Another important factor that increased the level of stress has also been the dynamic changes from stationary to e-learning [11]. However, the results of studies that assessed the impact of stress on the results of exams are inconclusive. One study has shown the lack of association between stress and the results of exams [12], while the other described associations between levels of stress and the lack of interpersonal relationships with peers, and consequently poorer exam results [13].

It is crucial to learn from the experiences to date; to make every effort to improve the quality of e-learning in the event of any further escalation of the ongoing pandemic [5, 14]. The search for safe solutions that allow medical students to obtain the appropriate practical competencies is also necessary. Though some recently published studies have shown that even highly practical skills, such as ultrasound, or basic surgical skills may be effectively taught using online courses [15–17].

Therefore, the aim of the study was to evaluate the perception of e-learning by students at Medical Universities in Poland.

## Material and methods

The anonymous survey addressed to medical students was placed on the Google Form platform and invitations to participate in the survey were sent to all Medical Universities nationwide. The survey was available from 30th November 2020 to 10th February 2021. This was a pilot study therefore the survey has not been validated.

The inclusion criterion for the respondents was studying medicine. There were no exclusion criteria.

The questionnaire included 23 questions: sociodemographic data (gender, age, place of residence, year of study), the assessment of various types of online and stationary classes, the assessment of prepared lecturers for classes, the assessment of the impact of e-learning on preparation to the profession and maintaining the quality of learning, the influence of e-learning on individual learning habits, advantages, disadvantages, and the limitations of e-learning.

The participants answered some of the questions with a Likert scale (1-very bad, bad, neutral, good, 5-very good).

The answers to the questions were recorded and only after answering the question could you move on to the next one. Multiple completions of the questionnaire by the same person were prevented.

Survey content:

Sociodemographic data: gender (female/male/non-binary/don’t want to specify), age, place of residence during the study before the outbreak of the COVID-19 pandemic (at family home/in a dormitory/ rented apartment), the place of residence during study during the COVID-19 pandemic (at family home/in a dormitory/ rented apartment), study mode during a pandemic (stationary/hybrid/I have stopped studying).

### Evaluation of teaching during the pandemic

What year of studies were you when the COVID-19 pandemic began?

What is the current form of class? (Lectures, seminars, laboratories, passing the course – answers - stationary/remote/hybrid/not applicable).

What is your attitude towards particular forms of remote learning? (Lectures, seminars, laboratories, passing the course – the answers on the Likert scale – from 1-very bad to 5- very good).

Has your university developed its platform for e-learning classes? (Yes/No).

What tools are used for remote learning at your university?

a. Microsoft Teams

b. Zoom

c. Genially

d. Class Dojo

e. Google Classroom

f. Kahoot

g. Quizlet

h. Educator

i. Khan Academy

j. Dzwonek.pl

k. Scholaris

What is your assessment of the preparation of teachers for classes during the COVID-19 pandemic? (The answer: It has got much worse to It has significantly improved) - answers on a Likert scale from 1 to 5

How do you rate the conduct of remote classes during the pandemic? (The answer: Very bad to Very good) - answers on a Likert scale from 1 to 5.

How do you rate the conduct of stationary classes during the pandemic? (The answer: It has got much worse to It has significantly improved) – answers on a Likert scale from 1 to 5.

How do you rate the requirements for passing the exams during the pandemic? (The answer: They have gone down a lot to They have made it very difficult) – answer on a Likert scale from 1 to 5.

Do you consider that the subject grade during the pandemic is equivalent to the grade obtained before the pandemic?

a. Yes, getting a passing grade/passing the exam is just as difficult

b. No, it is much easier to pass the exam now

c. No, it is much more difficult to pass the exam now

How will remote learning affect your preparation for the profession and initiation of a career?

a. Positively

b. Negatively

c. It will not affect my preparation

d. I don’t know

Do you think that the university did everything possible to maintain the level of education after the pandemic began? (Yes/No/ I have no opinion).

In the current situation, is it possible to maintain the quality of education and to teach the relevant material in remote/hybrid classes? (Yes/No/ I do not know).

Which of the following limitations did you experience and could have affected the quality of your education?

a. Adequate access to the internet

b. Quality of content taught and materials available

c. Conditions at home

d. Need to purchase or retrofit computer equipment

e. I have not experienced any restrictions

Has the pandemic affected the way you study?

a. I study more

b. I study less

c. I study with classmates less often

d. I spend more time finding materials on my own

e. I spend less time looking for materials on my own

f. My way of learning has not changed during the pandemic

Indicate what are the advantages of remote learning? (open question)

Please specify what are the limitations of remote learning? (open question).

What do you miss the most during hybrid/online classes? (open question).

### Statistical analysis

Statistical analyses were done with STATISTICA 13.0 PL (TIBCO Software Inc., Palo Alto, CA, USA) and R software (R Core Team (2013), R Foundation for Statistical Computing, Vienna, Austria, http://www.R-project.org/). A *p*-value below 0.05 was considered statistically significant. No data imputations were done in case of missing data. Nominal and ordinal data were expressed as numbers and percentages. Interval data with normal distribution were shown as the mean value ± standard deviation (SD). The distribution of variables was evaluated by the Anderson¬–Darling test and the quantile-quantile (Q–Q) plot.

## Results

### The characteristics of the respondents

Six hundred fifteen respondents fulfilled the questionnaires. The characteristic of respondents is presented in Table 1.

**Table 1:** Characteristics of the respondents

	*N* = 615	%
**Gender**		
Female	505	82.1
Male	105	17.1
Non-binary	5	0.8
**Age [years]**	23 ± 19	
**Place of residence**		
Rural	142	23.1
City < 50,000	111	18.0
City 50,000–100,000	69	11.2
City 100,000–250,000	81	13.2
City > 250,000	212	34.5
**Year of study**		
1	238	38.7
2	106	17.2
3	113	18.4
4	113	18.4
5	38	6.2
6	7	1.1

**Fig. 1:**
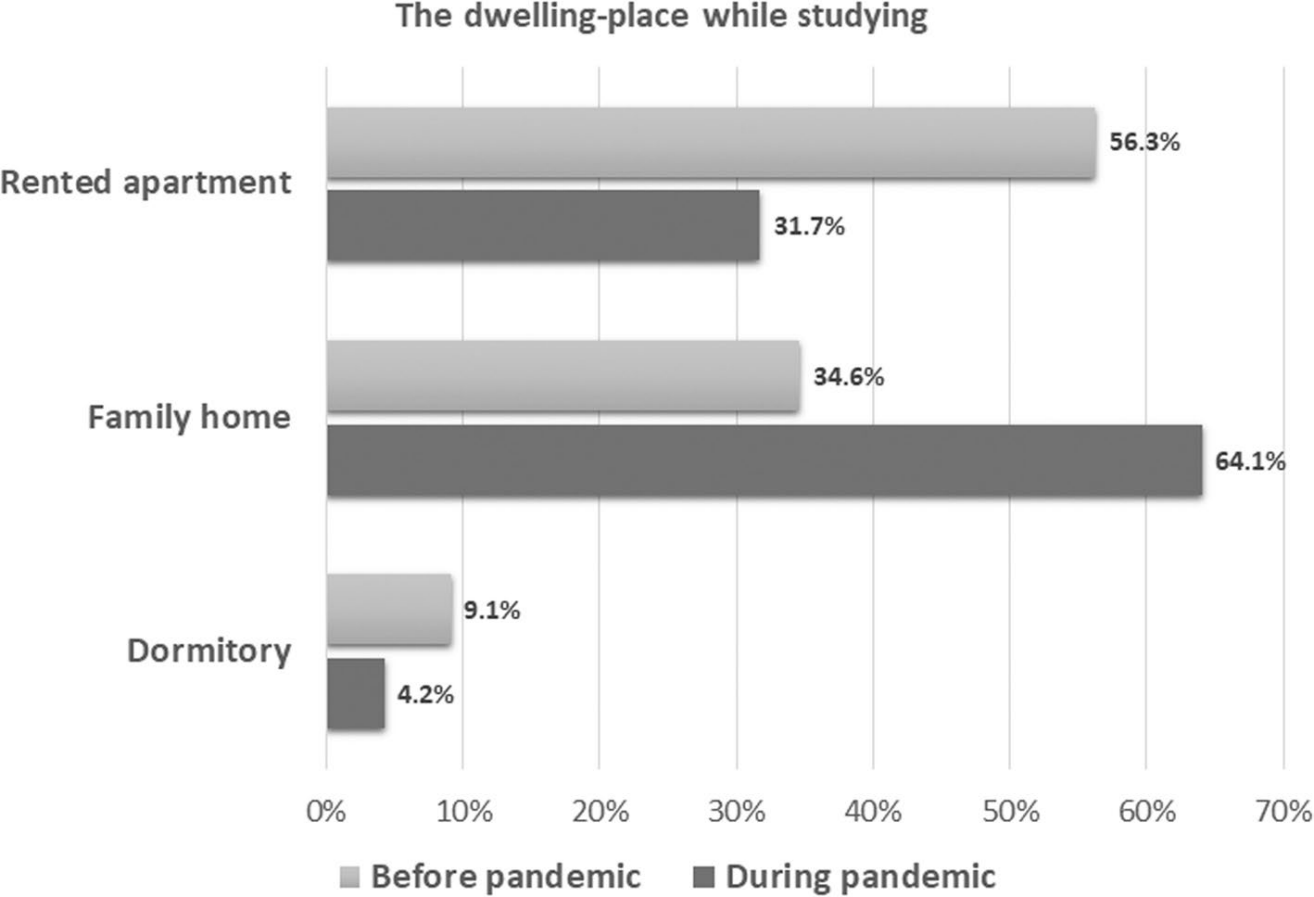
The dwelling place before and during the pandemic

After introducing the lockdown most of the students returned to their homes. The percentage of students renting apartments decreased from 56.3 to 31.7% and living in the dormitory from 9.1 to 4.2% (Fig. 1).

### Education during lockdown

Only, 2.1% of respondents indicated that their universities maintained a stationary form of education during the lockdown. 59.2% introduced a hybrid form and 38.7% an e-learning form only. The respondents reported that 96.1% of lectures, 85.5% of seminars, and 40.0% of clinical classes were conducted by e-learning. 71.5% of universities created their e-learning platforms. In addition, e-learning most often used Microsoft Teams, Zoom, Kahoot, and Quizlet platforms.

### Respondents’ assessment of education during the lockdown

The lectures conducted by e-learning were assessed as good and very good by 78.4% and seminars by 51.2% of respondents. While the clinical classes conducted by e-learning were assessed as bad and very bad by 62.9% of respondents. A similar percentage of respondents assessed stationary and e-learning classes as good and very good (30.8% vs. 30.7%). The preparation of lecturers for e-learning was assessed as good and very good by 23.6% of respondents and their commitment by 25.2% (Table 2).

**Table 2:** The perception of lecturers’ work, stationary and online classes

	Very good	Good	Neutral	Bad	Very bad	Not applicable
**The attitude towards different types of classes**						
online lectures	59.7	18.7	11.4	4.3	4.7	1.2
online seminars	25.2	26.0	23.2	15.5	7.3	2.8
online clinical classes	6.5	11.4	18.2	35.3	27.6	1.0
**The comparison of different types of learning**						
stationary classes	6.5	24.2	40.5	14.0	8.3	6.5
online ones	9.1	21.7	32.4	27.5	9.3	0
**The perception of lecturers’ work**						
the quality of preparations for online classes	6.0	17. 6	50.2	18.7	7.5	0
the perception of lecturers’ engagement	8.6	16.6	39.4	27.3	8.1	0

The results are presented according to the Likert scale in the survey (1-very bad, bad, neutral, good, 5-very good)

The lack of changes in requirements for completing the course was indicated by 30.2% of the respondents, higher by 43.9%, and lower by 25.9% of respondents. While 54.0% of the respondents indicated that it is easier to pass an exam during the pandemic, 26.2% that it has not changed, and only 19.8% that it is harder. However, 71.1% of the respondents indicated that the current situation would cause worse preparation for the profession, 11.7% reported that the current situation will not have an impact on preparations, and 3.6% indicated that it would get better.

27.3% of the students reported that they learn less during the pandemic and simultaneously 21.7% of the respondents spend more time finding adequate materials for the classes. Whereas 14.1% of them rarely learn with colleagues than before the pandemic, only 14.3% generally learn more during the pandemic (Table 3).

**Table 3:** Changes in learning habits and restrictions on education

Change of learning habits	%	Restriction on the education process	%
I learn less during the pandemic	27.3	quality of the content and available materials	26.9
I have to spend more time self-studying	21.7	the lack of contact with the lecturer	19.6
I learn rarely with colleagues than before the pandemic	14.1	inadequate Internet access	16.8
I learn more during online learning	14.3	home conditions	13.8
		purchase or retrofit computer equipment	4
		there was no restriction	9.7

The most frequently indicated limitations of e-learning were the quality of the content available materials (26.9%), restrictions in direct contact with the lecturer (19.6%), Internet connection (16.8%), and home conditions (13.8%). Only 4% of the students had to buy or retrofit computer equipment. Any other limitations were indicated by 9.7% of the respondents (Table 3).

In the opinion of 55.3% of the respondents, the Universities did not undertake all the measures they could to maintain a high level of education, 26.2% indicated that the University authority did everything they should, while 45.2% of respondents indicated that it is possible to maintain the quality of education during the pandemic, but a similar percentage did not agree with this (40.2%) and 14.6% had no opinion.

### Advantages and disadvantages of e-learning

**Fig. 2:**
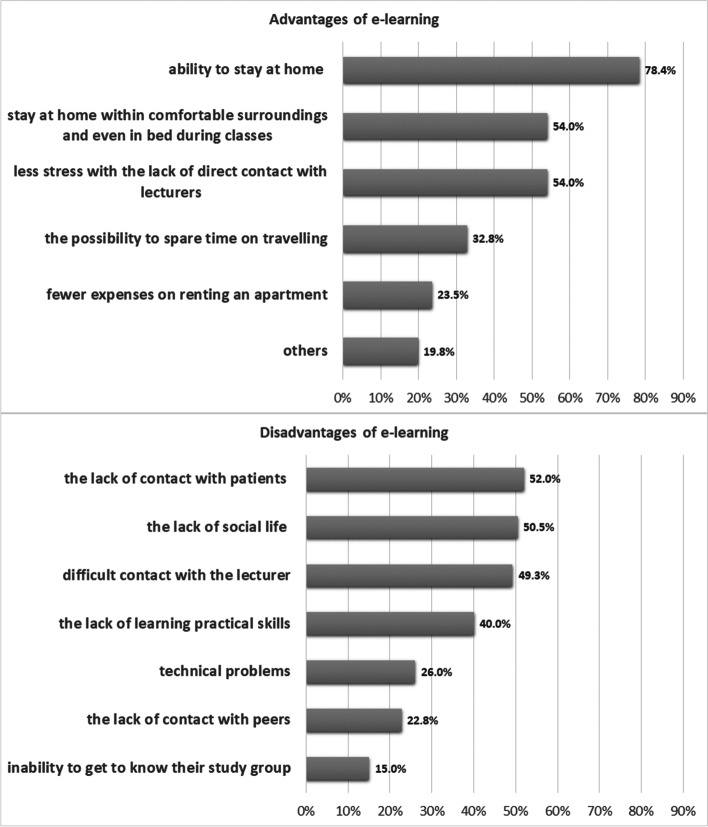
Advantages and disadvantages of e-learning

The most common indicated advantages of e-learning were the ability to stay at home (78.4%), comfortable surroundings (54.9%), and less stress with a lack of direct contact with lecturers (54.0%). While the most common indicated disadvantages were the lack of contact with patients (52.0%), the lack of social life (50.5%), and difficult contact with lecturers (49.3%) - (Fig. 2).

### Students’ expectations regarding e-learning

The most frequently indicated factors were better contact with the lecturer (38.7%), better Internet performance (33.2%), and better quality of the content and available material (33.0%). In addition, students suggested that the classes should be an interaction between the lecturer and the students in the form of analyzing clinical cases, making diagnoses, and suggesting treatment together.

## Discussion

Our study presents the results of an e-learning assessment by Polish students of Medical Faculties after about half a year of the introduction of epidemiological restrictions due to the COVID-19 pandemic. The first study was published 8 weeks after the introduction of the lockdown in March 2020 [14]. Studies have shown that the main problem associated with e-learning is the lack of interaction with the patients, especially as applies to students of the last 3 years of studies. Our study has also shown a low acceptance of laboratory and clinical classes in the form of e-learning. The lack of contact with patients may result in an insufficient gain of practical skills, an inability to communicate with patients, more misunderstandings, and inappropriate treatment [18]. This problem may be partially solved by inclusion in the e-learning programs with virtual patients. However, as shown in our study, despite the passage of time we did not apply this solution in Polish Universities. No alternative has been proposed either. However, it should be noted that even the best software with a virtual patient will not completely replace the contact and examination of a living patient. A more difficult, but feasible, the solution was the blended learning including theoretical classes in a remote form (using the e-learning portal and Teams communicator) and practical classes with the participation of patients in the appropriate sanitary regime, introduced in one Polish Medical University in the Department of Conservative Dentistry with Endodontics [19]. In the face of successive waves of the pandemic, it is necessary to consider combining classes with virtual patients and in hospitals carried out in smaller groups. This approach will increase the safety and effectiveness of practical classes. In addition, it may be a solution to the problems related to the enrollment of students, which is growing year by year, without a simultaneous and adequate increase in the base of clinical hospitals. This hypothesis is supported by previous experiences, in Chinese Medical schools, and online problem-based learning techniques introduced during the SARS pandemic remain in the curriculum until today [20]. However, according to the results of the study performed by Hayat et al. [21] to make e-learning more effective is necessary: to determine the rules and requirements for holding virtual classes which provide the necessary standards, holding special training courses for professors and students, technical support, collaboration and networking with other universities in their virtual education, use a variety of interactive software to actualize more student participation, creating web groups to discuss educational topics, providing and supporting desirable educational software, increasing and improving the speed of the Internet, granting free Internet packages to professors and students. It seems that all of this was missing at Polish Medical Universities during the first wave of the pandemic because in our study 55.3% of the respondents assessed the organization of the learning as not sufficient. But in the opinion of 45.2% of respondents, it is possible to maintain the quality of education during the pandemic. The main restriction for the quality of education process respondents indicated the quality of the content and available materials, the lack of contact with the lecturer, and adequate Internet access. Similar problems were indicated by medical students in another Polish study [22], however, a part of the students considered positive aspects of e-learning as indicating bigger student-friendliness and unlimited attention from teachers [22]. This indicated that lecturers’ involvement is a very important part of the quality of e-learning.

However, most of the students are eager to use modern techniques in their process of learning. It seems that this stems from their familiarity with the use of the Internet and utilizing online tools in learning. Similarly, in another Polish study, dental students showed a high degree of acceptance of teaching theoretical subjects using the e-learning method. Moreover, students would like to continue this model after the pandemic has ended [19]. This is also confirmed by the results of our study because we observed a high acceptance by students of lectures and seminars in e-learning form, while only 20.4% of medical and nursing students from India believe that e-learning can replace conventional teaching [23]. Thus, it seems that part of medical training can be conducted in e-learning form, but its effectiveness should be improved. It is confirmed by the data published long before the COVID-19 pandemic [3] and during the pandemic [19]. However, current [19] and earlier studies indicated that e-learning should constitute a supplement, not a replacement for traditional classes [24, 25]. The data assessing the effects of hybrid learning on examination performance, gaining practical skills, and long-term consequences in medical education are still missing. During the COVID-19 pandemic exams also take place online. Interestingly, studies published before the COVID-19 pandemic showed that a large number of medical students believe that e-exams are objective, have a high-quality standard, and prefer this form of exam [26–28]. However, the most important problems related to e-exams are authentication of the examinee’s identity and answer papers [29]. The very important observation in our study was the fact that 54% of respondents believe that online exams were easier than conducted previously even if it was only an online form of the test written in classrooms. Furthermore, respondents refused to answer the question on the reliability of participating in the exam and the use of other sources as help. This indicated a big problem with the reliability of the e-exams. Even though we have created a database of questions and a random selection of them in our Department, which is recommended in the literature [30], we observed much better exam results than when students took them in the classrooms. It would be worth carrying out a study where the same exam would be repeated in two forms, and the comparison of the results could compromise the actual knowledge of students. Especially since 27.3% of the respondents stated that they learn less during the pandemic.

According to the result of a previously published study [5], we observed that students expect direct contact with the lecturer. Moreover, in our study students suggested that the e-learning classes should be performed in the form of analyzing clinical cases, making diagnoses, and suggesting treatment together with the lecturer. In addition, students expect a greater commitment from lecturers during e-learning classes and more interactive classes. All of these show that students don’t want tapes or reading material, only real-time e-learning classes.

In addition, our study has shown that the extension of time students stay at home brings another problem associated with a lack of self-discipline without direct surveillance by lecturers. Though admittedly, a previously published study suggested that e-learning may make students more mature to gain knowledge on their own [31]. Other studies have shown that less face-to-face contact with lecturers was associated with worse exam results [32]. Our study did not find an answer to this aspect because the students refused to answer the question of being honest during the exam and the results were not available.

In our study, important negative factors associated with e-learning were the lack of contact and interactions with friends and the lack of social life. Other studies have shown that this may negatively affect mental health including anxiety development [33, 34]. However, in our study, similar to results obtained in the UK population, [5] more than half of the respondents felt less stressed over e-learning. The consequences of stress may result in high cortisol levels, memory impairment [35], and worse retrieval of information but are also associated with exhaustion, and distress [36]. All may contribute to worse academic performance. Monitoring the mental health of students should be introduced and support programs available [37, 38] to prevent the deterioration in academic performance due to the development of stress.

Our study has also shown that despite the time elapsed from lockdown and summer break in 2020, technical problems still limit the process of e-learning education. The Universities need to provide Internet connection and software that is reliable and sufficient for conducting lectures for several hundred students at one time to maintain the quality of connections and avoid the interruption of speech. However, it should be emphasized that students must also have the appropriate connection and equipment. The problems with Internet connections were also often indicated in the studies performed in the UK [5] and Poland [22]. In our study, 16.8% of respondents cited inadequate Internet access as a restriction to the educational process. While in the study performed in Saudi Arabia 64.6% of respondents indicated receiving inadequate Internet access [39].

Of interest, in our study, the majority of respondents cited a positive aspect of e-learning, as the possibility to stay at home and learn in comfortable surroundings. The results of a previous study showed that most of the students believe that e-learning helped them save time [40]. A part of the respondents stated that during the at-home study, their concentration was impeded by other household members. These results are partially consistent with the study performed in the US that showed studying at home in familiar conditions may result in better and more effective learning [14, 34, 41]. However, it should be remembered that not all students have a favorable home environment for studying. Therefore, for those students, e-learning may expose problems with time, space, and access to computer equipment in their homes [18, 23], and in some cases may even need technical support and e-learning training [18, 21]. It should be noted that a study from the US showed that a large role is played in e-learning by the student’s commitment, maturity, and independence [31, 42]. These factors were not analyzed in our study.

The main limitation of this study is the lack of indication of the University’s location. This stems from complete anonymity. In the opinion of the authors of this study, anonymity was supposed to increase the number of respondents, but it did not happen. The second limitation is the withdrawal of the question about the unfair practice of passing the exam due to the refusal by respondents to answer. The third limitation of the study was the lack of assessment of exam results.

## Conclusions

Students were highly accepting of lectures and seminars conducted in the form of e-learning, but not laboratory and clinical classes. The main problems in e-learning are the quality of the classes conducted and the Internet connection. The students expect e-learning classes to be conducted in real-time, with direct, face-to-face contact with the lecturer.

## Data Availability

The quantitative datasets supporting the conclusions of this article are included in the article. More detailed datasets are available from the corresponding author on request.

## Abbreviations

SARS-CoV-2: Severe acute respiratory syndrome coronavirus 2
COVID-19: Coronavirus disease 2019
